# Heat and Drought Stresses in Crops and Approaches for Their Mitigation

**DOI:** 10.3389/fchem.2018.00026

**Published:** 2018-02-19

**Authors:** Mouna Lamaoui, Martin Jemo, Raju Datla, Faouzi Bekkaoui

**Affiliations:** ^1^AgroBioSciences Division, University Mohammed VI Polytechnic, Benguérir, Morocco; ^2^Office Chérifien des Phosphates-Africa, Casablanca, Morocco; ^3^National Research Council Canada, Saskatoon, SK, Canada

**Keywords:** drought, heat, stress, crop, productivity, genomics, biotechnology, agronomy

## Abstract

Drought and heat are major abiotic stresses that reduce crop productivity and weaken global food security, especially given the current and growing impacts of climate change and increases in the occurrence and severity of both stress factors. Plants have developed dynamic responses at the morphological, physiological and biochemical levels allowing them to escape and/or adapt to unfavorable environmental conditions. Nevertheless, even the mildest heat and drought stress negatively affects crop yield. Further, several independent studies have shown that increased temperature and drought can reduce crop yields by as much as 50%. Response to stress is complex and involves several factors including signaling, transcription factors, hormones, and secondary metabolites. The reproductive phase of development, leading to the grain production is shown to be more sensitive to heat stress in several crops. Advances coming from biotechnology including progress in genomics and information technology may mitigate the detrimental effects of heat and drought through the use of agronomic management practices and the development of crop varieties with increased productivity under stress. This review presents recent progress in key areas relevant to plant drought and heat tolerance. Furthermore, an overview and implications of physiological, biochemical and genetic aspects in the context of heat and drought are presented. Potential strategies to improve crop productivity are discussed.

## Introduction

Drought and heat can reduce crop productivity and yields leading to lower income for farmers. Reduction in yield by as much as 40% was observed for maize and 21% for wheat at approximately a 40% water reduction (Daryanto et al., 2016). In the case of cowpea, an important crop in Africa, yield reduction can vary between 34 and 68% depending on the developmental timing of the drought stress (Farooq et al., 2017). Reductions in crop productivity will be further exacerbated by the impacts of climate change. The Intergovernmental Panel on Climate Change (IPCC) report concludes with unequivocal evidence that the air and ocean temperatures have warmed, and the concentrations of greenhouse gases have increased (IPCC, 2014). Both of these factors have direct influences on plant growth and crop yields (Bita and Gerats, 2013; Stocker et al., 2013). Between 1880 and 2012, land and ocean temperature data show an average global warming of 0.85°C based on multiple independently produced datasets (Stocker et al., 2013). The atmospheric concentrations of the greenhouse gases carbon dioxide (CO_2_), methane (CH_4_), and nitrous oxide (N_2_O) have also risen, with net emissions approaching 300 ppm in the recent years (Stocker et al., 2013). Climate change is impacting the earth's crust resulting in infrequent and erratic precipitations, elevated temperatures, and expansion of affected land areas under flood or water deficit. These adverse conditions are contributing to development of drought-prone areas and consequently on the plant growth and crop productivity. Though the impact of increased CO_2_ on crop yield is a debated topic with some researchers arguing that elevated CO_2_ will increase photosynthesis. However, Gray et al. (2016) showed that rising CO_2_ did not counteract the effect of severe drought on photosynthesis and yield.

Several independent studies have demonstrated the effects of increased temperature and water stress on crop yields. For example in Canada, the extreme events that occurred during 2001 and 2002 and the droughts and floods during 2010 and 2011 had a devastating impact on crop yield reducing by as much as 50% (Wheaton et al., 2008). Between 1980 and 2016, major USA disasters, exceeding a billion dollars each per year, indicates that when drought and heat are combined, they cause expanded agricultural losses of more than $220 billion (Oceanic Atmospheric Administration website, 2015).

In developing countries, the impact of heat and drought is equally significant. For example in Morocco, the economy is largely dependent on agriculture with approximately 40% of the labor force involved. In years of drought, crop productivity is significantly reduced and thus directly affects the livelihood of farmers and consequently the economy. Wheat is a staple crop where it is used for bread (haxaploid wheat) and for couscous (Durum wheat). The effect of drought is significant on wheat productivity. For example, cereal productivity was reduced in 2015–16 by approx. 50% from to 7.5M tons production on an average year to 3.4M tons in 2015–16. Sub-Saharan countries are also negatively affected in years with significant drought. Crop failure due to drought was reported in West Africa, with droughts between 1975 and 1985 resulting in a per capita food production decline of 25% (Epule et al., 2013).

We propose that despite the complexity of plant responses to heat and drought and the limited improvements so far achieved in mitigating the effects of these stresses, there are opportunities, highlighted in this review, to improve productivity of plants under these stresses.

## Drought vs. heat stress

At the whole plant level, all abiotic stresses induce a cascade of physiological and molecular events resulting, in some cases, in similar responses. Drought, high salinity and cold can all be exhibited as a physiological dehydration at the cellular level (Vinocur and Altman, 2005).

Drought stress takes place when soil and atmospheric humidity is low and the ambient air temperature is high. This condition is the result of an imbalance between the evapotranspiration flux and water intake from the soil (Lipiec et al., 2013). Heat stress is defined as the rise in soil and air temperature beyond a threshold level for a minimum amount of time such that permanent harm to plant growth and development occur. A detailed multi-location study highlights the impact of temperature effects on the yields. These adverse conditions are contributing to development of drought-prone areas and consequently on the plant growth and crop productivity of major crops (Zhao et al., 2017).

It is important that both drought and heat have to be considered together because their combined effect is higher than when taken individually (Dreesen et al., 2012). In general, abiotic stress including heat and drought are controlled by multiple genes and the underpinning mechanisms are more complex than other traits such as biotic stresses that are generally characterized by monogenic resistance. In addition, other abiotic and/or biotic stresses often have additive influence to heat and drought response making the study additionally challenging.

Water shortage and soil salinity undeniably represent major challenges facing productivity as they trigger oxidative, osmotic and temperature stresses (Reynolds and Tuberosa, 2008; Landi et al., 2017). Moreover, heat and water stress were also reported to be linked, as the reduced stomatal conductance and transpiration under these conditions may induce heat stress as leaf temperature rises (Król, 2013). Under field conditions, when water deficit and high temperature occurs simultaneously, growth and performance of the plants decline rapidly especially in tropical and sub-tropical environments (Wahid et al., 2007; De Boeck et al., 2015; Niinemets, 2015; Zandalinas et al., 2016, 2017).

Leaf structure is affected by higher temperature often causing development of thinner leaves with higher leaf area (Loveys et al., 2002; Luomala et al., 2005; Poorter et al., 2009). These gross morphological changes are underpinned with changes in leaf anatomy. Leaves which develop under water deficit generally have smaller cells and higher stomatal density (Tisné et al., 2010; Shahinnia et al., 2016). Wahid et al. (2007) reported comparable effects of high temperature and water deficit on cell density, but limited data are available with respect to leaf anatomy changes in response to high temperature.

## Physiological responses to heat and drought stresses

Plants have adapted dynamic responses to handle abiotic stresses at the morphological, physiological, and biochemical levels, allowing them to survive under variable environmental conditions (Huber and Bauerle, 2016). Plant physiological responses to drought and heat stresses can be classified into two distinct mechanisms. Avoidance mechanisms are mainly morphological and physiological adjustments that provide an escape to the water or heat stress, including increased root system, reduced stomatal number and conductance, decreased leaf area, increased leaf thickness, and leaf rolling or folding to lessen evapotranspiration (Sicher et al., 2012; Goufo et al., 2017). Cuticular wax biosynthesis, on the surfaces of the aerial plant parts, is also strictly associated with an adaptive response (Lee and Suh, 2013). Tolerance traits maintain tissue hydrostatic pressure, by cellular and biochemical modifications, mainly through osmotic adjustments (Khan et al., 2015; Blum, 2017).

Under these water challenging conditions, plants perceive stresses through various sensors involved in response signaling. These are transduced by various pathways in which many signaling and transcriptional factors play important and specific functions (Hirayama and Shinozaki, 2010). Moreover, some plants have built-in mechanisms to trigger hydraulic, chemical, or electrical long-distance signals to launch systemic stress responses. Under drought, a decrease in root hydraulic conductivity manifests to prevent water losses from the plant to the dry soil. High temperature can also increase root moisture loss to harmful levels (Parent et al., 2010).

Water transport within a plant occurs under tension as determined by soil water availability and the atmospheric vapor pressure deficit, creating turgor pressure within cells. Physiological adjustments that maintain turgor pressure are important under changing environmental conditions. Water transport in roots is affected by various components such as root anatomy, water availability and salts in the soil (Boursiac et al., 2005). All of these factors are influenced by the activity of aquaporins, which are integral membrane proteins that function as channels to transfer select small solutes and water (Maurel et al., 2008; Vandeleur et al., 2014).

Photosynthesis comprises various components, including the photosystems and photosynthetic pigments, the electron transport system, and CO_2_ reduction pathways. A stress-induced negative effect on any component in these systems may lead to a reduction in the overall photosynthetic performance. Studies have shown that photosynthetic efficiency and transpiration rates decrease under water, salt, and heat stress when applied individually or in combination (Arbona et al., 2013; Zandalinas et al., 2016). This is mainly a consequence of stress-induced stomatal closure but also can occur by other nonstomatal limitations such as decreased leaf expansion, leaf senescence and inappropriate functioning of the photosynthetic machinery (Wahid et al., 2007; Saibo et al., 2009; Rahnama et al., 2010). This latter effect situation is frequently attributed to the lower internal availability of CO_2_, along with the inhibition of key photosynthetic enzymes and ATP synthases (Zlatev and Lidon, 2012; Zandalinas et al., 2016). Both heat and water stresses are reported to decrease electron transport, degrade proteins, and release magnesium and calcium ions from their protein-binding partners (Wahid et al., 2007; Rexroth et al., 2011; Zlatev and Lidon, 2012; Zandalinas et al., 2016). Extended exposure to high temperature also triggers a decrease in chlorophyll content, increased amylolytic activity, thylakoid grana disintegration and disruption of assimilates' transport (Kozłowska et al., 2007).

Decreases in photosynthetic rate are directly linked to Water Use Efficiency (WUE), which is one of the most important parameters in crop response to osmotic imbalances. A number of reports have shown that decreases in net photosynthetic rate are associated with stomatal closure resulting in increased WUE (net CO_2_ assimilation rate/transpiration) (Ruggiero et al., 2017). Stomatal closure is thought to have a more inhibitory effect on transpiration of water than on CO_2_ diffusion into the leaf tissues (Sikuku et al., 2010). A widely employed tool for screening physiological traits associated with WUE or “Transpiration Efficiency” is the use of carbon isotope discrimination, which reflects both CO_2_ exchange and water economy. This type of tool is critical to assess phenotypic variation within a large breeding population (Ellsworth and Cousins, 2016). Recent advances in photosynthesis research may open new opportunities to address the relevant issues when crop plants face adverse drought and heat stress (Ort et al., 2015; Kromdijk et al., 2016).

The reproductive phase of development is the most sensitive stage to high temperature stress in several crops including wheat (Farooq et al., 2011; Shanmugam et al., 2013; Dwivedi et al., 2017), chickpea (Kalra et al., 2008; Devasirvatham et al., 2012; Kaushal et al., 2013), maize (Cairns et al., 2012), and Sorghum (Djanaguiraman et al., 2014; Singh et al., 2016). The reproductive processes involving pollen and stigma viability, pollination anthesis, pollen tube growth, and early embryo development are particularly vulnerable to heat stress (Giorno et al., 2013). However, in general, male reproductive tissues are much more sensitive at all stages of development to high temperature stress than female reproductive tissues (Hedhly, 2011).

## Biochemical responses to heat and drought stresses

**Figure 1:**
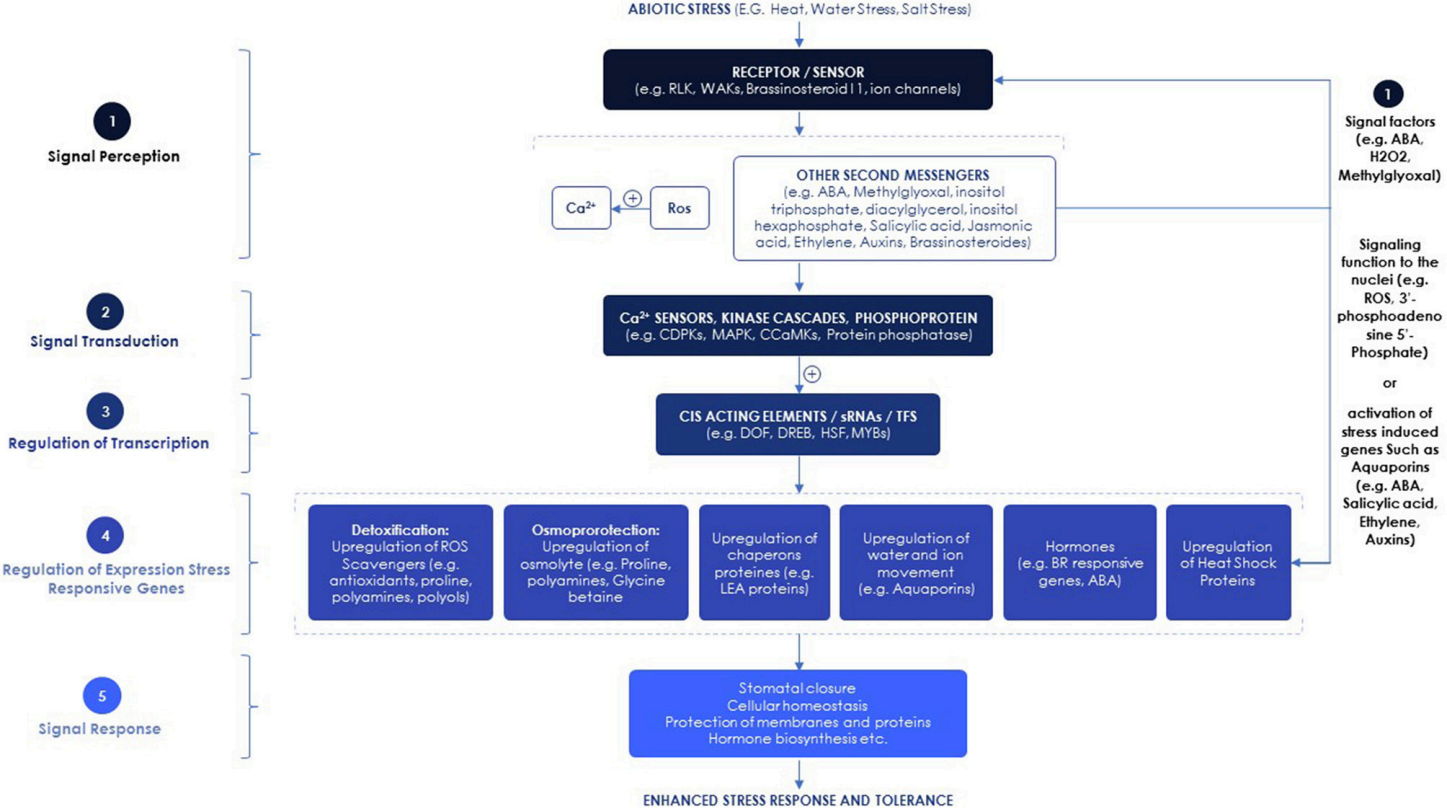
Signaling pathways involved in plant abiotic stress responses.

Signal perception through cell-surface receptors is a common feature of all living organisms. During stress conditions, reception of signals from the environment activates signaling cascades as a first step in a response (Figure 1) (Shulaev et al., 2008). Different types of receptors perceive various signals and stimuli from the environment. In the 1990s, the first receptor kinase protein, the Receptor-Like Kinase (RLK), was described in plants (Walker and Zhang, 1990; He et al., 1996). In vascular plants, a subfamily of RLKs known as WAKs (Wall-Associated Kinases) receives signals from the environment and other adjacent cells as a necessary step to activate appropriate signaling cascades (Shulaev et al., 2008). Since the finding of the first RLK, considerable efforts have been dedicated to characterizing additional specific receptor-kinase genes see in the Supplementary Data Table S1 for additional molecular factors involved in plant response to heat and drought stresses.

Stress perception is followed by the activation of systemic signaling cascades. Studies show that aquaporin proteins, key factors contributing to hydraulic conductivity, are regulated at the biochemical level by environmental stimuli with changes such as phosphorylation (Johansson et al., 1998), cytoplasmic pH and calcium (Gerbeau et al., 2002; Alleva et al., 2006) or re-localization to intracellular compartments (Boursiac et al., 2008).

Plant hormones, have also been shown to be implicated in root and shoot long-distance signaling and the control of hydraulic conductivity. Abscisic acid (ABA) is the most critical hormone involved in regulating tolerance to abiotic stresses such as drought, salinity, cold, heat and wounding (Zhang et al., 2006; Lata and Prasad, 2011). ABA has long been acknowledged as a major chemical root-to-shoot stress signal (Schachtman and Goodger, 2008), inducing inhibition of leaf expansion and short-term responses like stomatal closure. ABA is involved in the regulation of systemic responses to abiotic stress before there are any detectable changes in leaf water or nutrient status (Bauer et al., 2013; Suzuki et al., 2013). Osmotic stress results in the synthesis or catabolism of several other growth regulators, including auxin, cytokinins, ethylene, gibberellins, brassinosteroids, jasmonic acid and other factors (eg. nitrogen, pH) that have been shown to be involved in the regulation of physiological processes through their action as signal molecules in signaling networks (Nakashima and Yamaguchi-Shinozaki, 2013; Mittler and Blumwald, 2015; Verma et al., 2016).

Increased intracellular Ca^2+^ levels are also induced under stress conditions by several signal molecules such as: inositol trisphosphate, inositol hexaphosphate, diacylglycerol and Reactive Oxygen Species (ROS) (Hirayama and Shinozaki, 2010). Calcium binding proteins, that function as Ca^2+^ sensors, perceive the elevated Ca^2+^ levels (Kudla et al., 2010), which can lead to the activation of calcium dependent protein kinases. The activated kinases or phosphatases can phosphorylate or dephosphorylate specific transcription factors (TFs), thus regulating the expression levels of stress-responsive genes (Reddy et al., 2011). The activated Ca^2+^ sensors can also bind to cis-elements of major stress-responsive gene promoters or can interact with DNA-binding proteins regulating these genes, resulting in their activation or suppression (Kudla et al., 2010; Reddy et al., 2011).

An early heat stress response was reported to induce Ca^2+^ influx and cytoskeletal restructuring, which causes the upregulation of mitogen activated protein kinases and calcium dependent protein kinase signaling cascades (Wahid et al., 2007; Ashraf and Harris, 2013). This signaling cascade leads to production of antioxidants and compatible osmolytes (for osmotic adjustment) and the expression of heat shock proteins. The main impacts of heat stress are protein denaturation, instabilities in nucleic acids and cytoskeletal structure, increased membrane fluidity, inactivation of the synthesis and degradation of proteins, and loss of membrane integrity (Howarth, 2005; Wahid et al., 2007). Severe cellular injuries or loss can occur at moderately high temperatures, after long-term exposure, or after extremely short-term exposure to very high temperatures (Wahid et al., 2007). This may reduce ion flux and lead to production of ROS and other toxic compounds which severely affect plant growth (Howarth, 2005). Expression of heat shock proteins as well as other types of protective proteins is an effective adaptive strategy under conditions of high temperatures exposure—with the expression of said protective proteins often correlating with stress tolerance (Wahid et al., 2007), photosynthesis and WUE (Camejo et al., 2005), membrane stability (Ahn and Zimmerman, 2006), and maintenance of cellular hydration (Wahid and Close, 2007).

Various abiotic stresses induce overproduction of ROS, which are extremely reactive and toxic, causing damage to proteins, lipids, carbohydrates and DNA and ultimately resulting in oxidative stress (Zlatev and Lidon, 2012). Oxidative stress is commonly counteracted by two different processes, prevention or avoidance of ROS formation, including detoxifying/scavenging enzymes and several antioxidants to handle the toxic effect of ROS (Mittler, 2002; Gill and Tuteja, 2010). Concomitantly with ROS, plants also produce 2 to 6-fold higher methylglyoxal under abiotic stress (Yadav et al., 2005), which is a very reactive cytotoxic compound produced through different enzymatic and non-enzymatic reactions. Methylglyoxal damages cellular function and can even destroy DNA (Hasanuzzaman et al., 2017). Methylglyoxal is detoxified through the glyoxalase system composed of glyoxalase I and glyoxalase II, that catalyze methylglyoxal to D-lactate via reduced glutathione as a cofactor (Yadav et al., 2005).

Recently, new retrograde signals have been reported such as the metabolite 3′-phosphoadenosine 5′-phosphate that has been shown to accumulate during high light and drought, moving from chloroplast to nucleus to regulate ABA signaling and stomatal closure during the oxidative stress. This results in drought tolerance and the activation of the high light transcriptome (Pornsiriwong et al., 2017).

TFs play important roles in stress tolerance. Many abiotic stress-related genes and TFs have been isolated from different plant species and overexpressed in transgenic plants to improve stress tolerance (Table 1). The stress-inducible TFs include members of the DREB, ERF, WRKY, MYB, bHLH, bZIP, DOF, and NAC families (Shinozaki et al., 2003) (Figure 1). For example, the overexpression of Cycling Dof Factor 3 in *Arabidopsis* enhanced drought, cold and salt tolerance (Corrales et al., 2014, 2017), along with the up-regulation of a set of genes involved in cellular osmoprotection and ROS homeostasis (Corrales et al., 2017). In a recent review, Kulkarni et al. (2017) discussed the roles of other transcriptional regulators including ZFPs and EAR-motif containing repressors in abiotic stress regulation.

**Table 1:** Summary of genes that were shown to have a role in heat and drought tolerance in wheat and maize.

**Gene/origin**	**Function/Mechanism**	**Type of promoter/Expression**	**Trait**	**Crop**	**Author**
DREB1A/*A. thaliana*	TF	rd29A gene promoter/stress-inducible gene, upregulated	Improved drought salt and freezing tolerance	Wheat	Pellegrineschi et al., 2004
Hsf6A (heat shock factor)/wheat	TF	Barley *HVA1s* promoter/Drought inducible, upregulated	Improved thermo tolerance	Wheat	Xue et al., 2014
AtHDG11/*A. thaliana*	TF	Actin1 promoter/overexpression	Improved drought tolerance	Wheat	Li et al., 2016
TOR/*A. thaliana*	Signaling Factor	CaMV35S promoter/overexpression	Improved drought tolerance	Wheat	Datla et al., 2014 (Patent application and unpublished results)
EF-Tu/maize	Elongation factor/chaperone like activity	Maize ubiquitin 1 promoter/overexpression	Improved thermos tolerance	Wheat	Fu et al., 2008
Stress-responsive NAC gene/rice	TF	Maize ubiquitin 1 promoter/overexpression	Enhanced tolerance to drought and salt stresses	Wheat	Saad et al., 2013
HVA1/barely	Group 3 LEA - HVA1	Maize ubi1 promoter/overexpression	Improved drought tolerance and field evaluation for drought tolerance	Wheat	Sivamani et al., 2000; Bahieldin et al., 2005
P5CS/*Vigna aconitifolia*	Proline Biosynthesis	Stress-induced promoter complex—AIPC/upregulation	Increased tolerance to water deficit	Wheat	Vendruscolo et al., 2007
Phosphoenolpyruvate carboxylase (PEPC)/maize	C4, CAM and the citric acid cycles	Maize PEPC promoter/overexpression	Improved yield and drought tolerance	Wheat	Qin et al., 2016
OsMYB55/rice	TF	Maize ubiquitin Ubi1 promoter/overexpression	Increased drought and heat stress tolerance	Maize	Casaretto et al., 2016
ZmNF-YB2/maize	TF (Nuclear factor Y B subunit 2)	Rice actin 1 constitutive promoter/overexpression	Enhanced drought tolerance and photosynthetic capacity	Maize	Nelson et al., 2007
ZmPIS gene/maize	Precursor of signal molecules	Maize ubiquitin promoter/overexpression	Enhanced drought tolerance	Maize	Liu et al., 2013
NPK1/tobacco	Protein kinase	Constitutive 35 S promoter/overexpression	Enhanced drought tolerance	Maize	Shou et al., 2004
CspA, CsB/bacteria	RNA chaperones, cold shock protein	Rice actin1 promoter/overexpression	Improved kernel yield under water limiting conditions in the field	Maize	Castiglioni et al., 2008
1-aminocyclopropane-1-carboxylic acid synthase 6/maize	Ethylene Biosynthesis	Maize ubiquitin promoter/downregulation	Improved grain yield under drought stress conditions in the field	Maize	Habben et al., 2014
ZmARGOS1/maize	Down regulator of ethylene response and modulator of ethylene signal transduction	Maize ubiquitin promoter/overexpression	Drought tolerance enhancement in the field	Maize	Shi et al., 2015
ZmARGOS1/maize	Down regulator of ethylene response and modulator of ethylene signal transduction	Genetic editing/downregulation	Drought tolerance enhancement in the field	Maize	Shi et al., 2017
LOS5/*A. thaliana*	Cofactor sulfurase gene	“Super” promoter (manopine synthase)/overexpression	Enhanced drought tolerance	Maize	Lu et al., 2013
betA gene/*E. coli*	Biosynthesis of glycine betaine	CaMV35S promoter/overexpression	Enhanced drought stress tolerance	Maize	Quan et al., 2004
Trehalose-6-phosphate phosphatase (OsMADS6)/rice	Sucrose metabolism	OsMads6 promoter/overexpression	Enhance yield under well-watered and water stressed plants in the field	Maize	Nuccio et al., 2015

The production of a wide range of metabolites of low molecular mass can prevent the detrimental change in cellular components and restore homeostasis. These include soluble carbohydrates such as glucose and fructose, amino acids and a variety of sugars and sugar alcohols (Vinocur and Altman, 2005; Szabados and Savouré, 2010; Arbona et al., 2013; Morales et al., 2013). The accumulation of these compounds has been linked to abiotic stress tolerance and maintenance of cell turgor, protection of protein structures and stabilization of cellular membranes as cells dehydrate (Arbona et al., 2008, 2013; Zlatev and Lidon, 2012). Among amino acids, proline is the main effector contributing to osmotic adjustment in response to different abiotic stress conditions (Kaplan and Guy, 2004; De Campos et al., 2011; Morales et al., 2013; Sinay and Karuwal, 2014; Zandalinas et al., 2017). Therefore, the synthesis and accumulation of proline has been considered a tolerance trait for a significant amount of time (Janská et al., 2010).

Glycine betaine also plays an important role in abiotic stress tolerance in some plant species. Genes associated with glycine betaine synthesis in higher plants and microbes have been transferred into plants such as maize, which do not accumulate glycine betaine or enhance the level of synthesis upon stress (Quan et al., 2004). However, the mechanism of action of glycine betaine is unclear. Using a transgenic system, Su et al. (2006) observed that the apparent effects of glycine betaine biosynthesis on stress tolerance may be attributed to protective effects other than changes to the cellular osmotic balance.

Secondary metabolites are also essential compounds for plant acclimation and persistence under fluctuating environmental conditions and include: coumarins, lignin, anthocyanins, flavonoids and tannins (Fraser and Chapple, 2011). In citrus plants, increased levels of secondary metabolites such as phenylpropanoid, precursors of lignins, flavonols, and flavones were observed in response to drought and temperature stresses (Zandalinas et al., 2017).

## Agronomic strategies for improving tolerance to drought and heat

Implementation of crop management practices can potentially alleviate the harmful effects of drought and heat stresses and include: soil management and culture practices, irrigation, crop residues and mulching, and selection of more appropriate crop varieties.

Under heat stress, the application of macronutrients such as K, Ca and micronutrients like B, Se, and Mn, which are known to modify stomatal function, can help activate the physiological and metabolic processes contributing to preserving high water potential in tissues thereby increasing heat stress tolerance (Waraich et al., 2012). The application of nutrients such as N, K, Ca, and Mg was also reported to reduce toxicity to ROS by increasing the concentration of antioxidant enzymes in plant cells (Waraich et al., 2012). On the other hand, several studies have shown that the application of fertilizer has no significant effect on drought stress and an optimal soil moisture content is required since water is critical for the mobility and metabolism of these nutrients (Lipiec et al., 2013).

The effects of drought have been alleviated through exogenous silicon (Si) application in wheat and rice (Gong et al., 2005; Gautam et al., 2016). Plants treated with Si displayed higher antioxidant activities (Gong et al., 2005; Ma et al., 2016), higher amounts of photosynthetic pigments (Gong et al., 2005), and expression changes of genes implicated in the ascorbate-reduced glutathione cycle, flavonoid biosynthesis and antioxidant response (Ma et al., 2016). Seed priming with Si was also efficient in the protection of maize plantlets from alkaline stress (Abdel Latef and Tran, 2016).

Attention has also been focused on the application of plant growth regulators known to be involved in the response to stress. Among the plant growth substances, salicylic acid, cytokinin and ABA have been reported to play a key role in drought tolerance. Under water stress conditions, plant growth regulator treatments can significantly increase the water potential and the chlorophyll content (Zhang et al., 2004). Exogenous application of ABA increased soybean yields under water deficit conditions (Zhang et al., 2004). New ABA formulations are currently available for commercial growers to delay drought-induced wilting symptoms and improve drought tolerance (Barrett and Campbell, 2006; Sharma et al., 2006; Blanchard et al., 2007; Huang et al., 2008; Waterland et al., 2010). Recent research has investigated the use of concentrated ABA or ABA analogs to maintain the marketability of horticulture crops by reducing drought stress symptoms (Monteiro et al., 2001; Sharma et al., 2006; Blanchard et al., 2007; Kim and van Iersel, 2008). ABA application during spring or summer also reduces transpiration in potted miniature rose (*Rose hybrida* L.) and results in better flower longevity (Monteiro et al., 2001). Perego et al. (2014) used another strategy to mitigate yield losses by bringing forward the date of sowing of maize in order to prevent heat stress during the flowering stage.

## Genetics and genomics approaches

Conventional plant breeding has had limited success in mitigating the effects of abiotic stress on plant productivity. This may be due to the complexity associated with traits controlled by a number of genes present at multiple quantitative trait loci (QTL) (Parmar et al., 2017). However, there are cases of successes in conventional breeding for improved heat and drought tolerance traits. For example, Haley et al. (2007) developed a drought tolerant variety of wheat referred to as “Ripper.” This variety performed well with superior grain yields under non-irrigated conditions in Colorado. The variety also has superior milling and bread-baking quality. Badu-Apraku and Yallou (2009) developed maize varieties that had superior yield compared to the control varieties grown under drought conditions. These maize cultivars also performed well under biotic stress conditions. The use of quantitative traits locus markers in breeding programs with marker-assisted backcrossing and marker-assisted recurrent selection strategies have also had positive results (Mir et al., 2012). QTLs from wild emmer wheat were introgressed through marker-assisted selection, to improve drought resistance in elite durum (*T. turgidum* ssp. durum) and bread (*T. aestivum*) wheat cultivars (Merchuk-Ovnat et al., 2016). Three of the introgressed QTLs were confirmed, two in the durum wheat background and one in bread wheat. In most cases, the QTL x environment interaction was validated under drought with regard to grain yield and biomass improvement. Tahmasebi et al. (2016) used a recombinant inbred line population to map QTLs under well-irrigated, heat, drought, and a combination of drought and heat stress conditions for two years. They identified a QTL that explained up to 19.6% variation in grain yield in the drought, heat, and combined stress trials. The authors proposed that the marker could be used as a candidate for validation in other populations and identifying superior allelic variations in wheat cultivars to increase the efficiency of selection of high yielding lines adapted to heat and drought stress conditions.

Despite challenges due to genome size and polyploidy of crops such as wheat, significant progress has been made in genome sequencing, annotation and functional characterization of important genes (Uauy, 2017). Clavijo et al. (2017) have recently generated a new wheat whole-genome shotgun sequence assembly using a combination of optimized data types and an assembly algorithm intended to deal with huge and complex genomes. They identified 104,091 high-confidence protein-coding genes and 10,156 noncoding RNA genes. Recently, Luo et al. (2017) have sequenced the genome of the progenitor of the wheat D genome (*Aegilops tauschii*). The genome sequence data of both wheat and Aegilops should permit identification of structural variants, and assist with the annotation of gene models including those involved in complex traits such as heat and drought.

In addition to protein coding genes, some miRNAs are functionally preserved across plant species and have been shown to be regulated by drought stress. In drought-resistant wild emmer wheat, miR166 was shown to be down-regulated under drought stress (Kantar et al., 2011). Recently, Huang et al. (2017) have identified a long non-coding miRNA gene that controls β-diketone wax formation; these waxes play an important role in drought tolerance acting to minimize water loss. These observations propose that targeted miRNA-based genetic modifications have the potential to improve drought tolerance in cereal crops (Ferdous et al., 2015).

Genomic studies of species that are extremely drought tolerant such as desiccation tolerant (DT) or resurrection plants may serve as models for designing crops with enhanced drought tolerance (Giarola et al., 2017). Desiccation tolerance is common in seeds and other organisms, but only some angiosperm species possess vegetative desiccation tolerance that have evolved due to ecological constraints (Giarola et al., 2017). *Xerophyta viscosa* is a monocotyledonous plant species closely related to cereal crops. Therefore, it is an ideal model for understanding extreme dehydration in cereals. Reactivation of vegetative DT is based on the presence of genes associated with DT in reproductive structures, such as seeds, and hence the genomic information for DT was redirected toward vegetative tissues (Costa et al., 2017). Costa et al. showed that ABI5 may be a regulator of expression of the *LEA4* family which may be an important factor in the longevity of *X. viscosa* in the dry state. These researchers also identified two structural orthologs of ABI3, a major regulator of seed maturation and DT along with the majority of the ABI3 regulated genes in leaves. The fact that LEA4 and ABI3 are involved in both drought tolerance and seed desiccation tolerance shows that these processes share similar mechanisms.

Epigenetics involves the study of gene expression changes caused by modifications of DNA structure without alterations to the nucleotide sequence. The modifications include changes in the methylation state of DNA, modification of histones and the expression of non-coding RNA. Epigenetics could be a potential new source for trait variation applicable for plant breeding (Mirouze and Paszkowski, 2011). Epigenetic control over plant response to stress is a complex phenomenon. Epigenetic modifications not only occur during plant exposure to stress, but can also establish epigenetic changes, resulting in modified gene expression, that can persist over several generations (Boyko and Kovalchuk, 2008). Zhu et al. (2008) showed that an imperfection in the deacetylase-like protein HOS15 produced an abnormal reaction to ABA and abiotic stress. Saez et al. (2008) identified SWI3B (a homolog of a chromatin remodeling complex) as one of the targets of an ABA-related PP2C, HAB1. These observations suggest that ABA and/or abiotic stress signaling modifies gene expression profiles and developmental programs through the modification of epigenetic status to cope with the stresses (Hirayama and Shinozaki, 2010).

## Application of transgenic and genome editing tools and technologies

Understanding the mechanisms of abiotic stress damage is critical for the development of tolerant plant species and varieties. Application of transgenic-based approaches could help to introduce desirable abiotic stress tolerance traits into crop varieties. Toward this end, several studies have used genes and TFs associated with abiotic stress tolerance as target genes in the application of biotechnological strategies to develop drought tolerant plants.

Despite the benefits of commercial genetically engineered (GEn) plants (National Academies of Sciences, 2017) and the promising results shown with numerous GEn prototypes addressing abiotic stresses in crops, the broader application of this technology remains a major challenge because of the negative public perception regarding the intentional introduction of genes into plants, particularly in Europe. It is a complex issue and the lack of acceptance of GEns can be for different reasons. It is not the intent of this review to provide an in-depth discussion on this topic. However, given the significant potential of improving heat and drought through GEn, it is relevant to review some aspects of the applications of this potential technology.

Phytohormones are potential targets for genetic manipulation to obtain abiotic stress tolerant crops. Overexpression of ABA-pathway-related TFs, imparts an ABA-hypersensitive response and also improves the osmotic stress tolerance in transgenic plants (Abe et al., 2003; Gao et al., 2011). Similarly, transgenic plants overexpressing RD26, a stress-inducible NAC TF, have also shown high ABA-sensitivity and thus an up-regulation of ABA and stress-responsive genes (Fujita et al., 2004). Under moderate drought stress, during the flowering period, the yields of transgenic canola overexpressing a farnesyltransferase protein were significantly higher comparatively to the control (Wang et al., 2005). The overexpression of TFs that control root architecture induced drought tolerance in rice and transgenic *Arabidopsis* plants by promoting root growth and thus enhancing WUE (Redillas et al., 2012; He et al., 2016). Other TFs linked to WUE, such as those stimulating wax deposition in cuticle and suberin deposition (Legay et al., 2016), and other regulators able to modulate entire pathways, could be used for the same objective to activate stress-response genes and enhance tolerance (Casaretto et al., 2016; Landi et al., 2017).

Engineering of the glyoxalase pathway has been reported to enhance tolerance to abiotic stress in different plant species. Upregulation of both glyoxalase I and glyoxalase II and their overexpression in plant species revealed enhanced tolerance to various abiotic stresses including salinity, drought, metal toxicity, and extreme temperature (Singla-Pareek et al., 2003, 2008; Bhomkar et al., 2008; Lin et al., 2010; Tuomainen et al., 2011; Wu et al., 2013; Alvarez-Gerding et al., 2015; Hasanuzzaman et al., 2017).

Table 1 shows examples of GEn applications with drought and/or heat tolerance improvement in wheat and maize compared to non-transformed plants. These observations confirm that the genes introduced into plants have a role in stress tolerance. Generally, the transformed lines compared to the controls show less transpiration, higher metabolism of ROs, increased production of protective molecules such as proline, increased root mass and improved photosynthesis rate. These changes contribute to a higher yield of the transformed plants under abiotic stress compared to the control plants. It is worth noting that with the exception of one case (Habben et al., 2014), all the examples shown in Table 1 involve overexpression of the introduced gene. Over time, we notice that genes from the same species are used in transformation in combination with a different promoter (cisgenic) rather than using a gene from a different species (transgenic). This may be done so that there is a better public acceptance of GEn technology.

Demonstration of the beneficial effect of the genetic transformation under field condition compared to greenhouse conditions provides stronger evidence for the possible economic benefits of the GEn technology. Several studies are limited to greenhouse trials because of the regulatory requirement and additional cost for the confined trail with GEn plants. Nevertheless, evidence for improved abiotic stress tolerance has been shown under field conditions in several cases (Bahieldin et al., 2005; Nelson et al., 2007; Castiglioni et al., 2008; Nuccio et al., 2015).

Thus by applying GEn technologies, there is significant potential to improve drought and heat tolerance in important crops like wheat and corn. However, because of the stringent regulations for field trials of GEn plants it will be difficult to fully validate these observations toward improving crop productivity. Genome Editing (GEd) technologies may provide an acceptable alternative to address this issue. The advantage of GEd is that genetic modifications achieved using this technology produce minor genome changes that are equivalent to what is considered non-GEn, for example mutations generated through chemical and physical agents that are widely used in plant breeding. In GEd technologies, the targeted DNA sequence modifications are achieved by application of sequence-specific nucleases that create double-strand breaks in the target genomic loci selected for editing. The main methods used for gene editing in plants are Zinc Finger (ZF) nucleases, transcription activator–like effector nucleases (TALENs) and clustered, regularly interspaced, short palindromic repeats (CRISPR) (Voytas, 2013). Various strategies of GEd could be used to improve agronomic traits; for example, introduction of a premature stop codon to disable protein function or changes to a gene promoter motif to modulate gene expression. Wang et al. (2014) used TALEN and CRISPR-Cas9 technologies in hexaploid bread wheat to introduce targeted mutations in the three homoeoalleles that encode MILDEW-RESISTANCE LOCUS (MLO) proteins. Alternatively, to increase gene expression, Piatek et al. (2015) used a synthetic transcriptional repressor and activators as endogenous TFs to activate transcription of an endogenous genomic target. A better understanding of the physiological response to heat and drought stresses and connection with other biological processes will assist in the design of the most efficient means for GEd of plants toward improved stress resistance.

## Future perspectives/conclusions

Plants have evolved sophisticated adaptive mechanisms to withstand diverse and complex abiotic stresses. With the advent of new technologies such as genomics and genetic transformation, significant progress has been made in understanding these complex traits in higher plants. However, the commercial application of positive research outputs requires further validation of products or prototypes in the field. There are further opportunities for research discoveries from new emerging frontiers such as epigenetics, GEd and plant interactions with the soil microbiome.

For example, the area of soil microbiome research offers opportunities for improving biotic and abiotic stress in crops. Plant mechanisms to escape from drought and/or heat stresses can be mediated by the microbes surrounding a plant, particularly the roots, and are associated with various plant developmental stages, physiological cascades, and biochemical and molecular reactions occurring at the cellular, tissue, or whole plant level. Advances made with the application of novel molecular and genomic tools and techniques have opened research avenues into plant microbiota and these encouraging developments are allowing the exploration of the biological functions of a wide diversity of microorganisms both inside and outside of plant host tissues.

Significant advancements in crop genome characterization and the optimization of genome editing technology in crops have and will continue to advance our understanding and capabilities toward development of stress tolerant crops. Ultimately, genome editing or transgenic approaches need to be combined with efforts using conventional and marker-assisted breeding activities to achieve the desired improved varieties. Further, it is important to take into account climate change models, which differ geographically, to guide breeding programs in target trait identification for selection and identification of new adapted germplasm (Harrison et al., 2014). These efforts will lead to tangible practical outcomes that may help mitigate the effects of climate change, especially with respect to drought and heat stresses, and will contribute to improved crop productivity and food security, particularly in areas such as Africa.

## Author contributions

FB coordinated the review and drafted the abstract, the introduction and conclusions. ML contributed with the physiological and biochemical sections and editing the references. MJ contributed with the microorganisms sections. FB and RD contributed with the genetics and genomics components. FB, ML, MJ, and RD contributed to the revision of the whole manuscript.

### Conflict of interest statement

The authors declare that the research was conducted in the absence of any commercial or financial relationships that could be construed as a potential conflict of interest.

## Abbreviations

ABA: Abscisic acid
DREB: dehydration-responsive element-binding proteins
DT: Desiccation tolerance
GEd: Genome Editing
GEn: Genetic Engineering
LEA protein: late embryogenesis abundant protein
ROS: reactive oxygen species
TF: Transcription Factor
WUE: Water Use Efficiency.
