# Supplementary material for: Heat and Drought Stresses in Crops and Approaches for Their Mitigation
Source: Front Chem. 2018 Feb 19;6:26. doi: 10.3389/fchem.2018.00026 (PMC5827537; doi:10.3389/fchem.2018.00026)
Supplement: Supplementary file 1 [file DataSheet1.DOCX]

**Supplementary Table S1: Key molecular factors involved in plant response to heat and drought stresses.**

| **Molecules** | **Function** | **Author** |
| --- | --- | --- |
| Receptor kinase protein | Signal perception - Brassinosteroid | Ronald, 1998; |
|  | Signal perception - CLAVATA1 | McCubbin and Kao, 2000; |
|  | Signal perception - Pto | Schumacher and Chory, 2000; |
|  | Signal perception - S-locus | Sessa and Martin, 2000; |
|  | Signal perception - Xa21 | Clark, 2001 |
| Aquaporin | H_2_O_2_ accumulation in response to ABA or pathogen-associated molecular pattern | Rodriguez et al. 2017 |
| Abscisic acid | Activation of many stress-related genes | Nakashima et al. 2012; Mittler and Blumwald, 2015; Sah et al. 2016 |
|  | Hydraulic conductivity regulation and expression mainly aquaporin | Aroca et al. 2006; Kudoyarova et al. 2011 |
|  | Increase of cytosolic Ca^2+^ | Kohler and Blatt, 2002; Verma et al. 2016 |
|  | Regulating abiotic stress tolerance | Zhang et al. 2006; Lata and Prasad, 2011 |
|  | Root-to-shoot stress signal | Schachtman and Goodger, 2008 |
|  | Enhances synthesis of protective proteins and osmolytes synthesis | Fujii et al. 2011 |
| Salicylic acid and Ethylene | Interaction with ABA | Fedoroff, 2002; Fujita et al. 2006; Grant and Jones, 2009; Pieterse et al. 2009; Verma et al. 2016 |
|  | Aquaporin regulation | Boursiac et al. 2008; Chervin et al. 2008; Tungngoen et al. 2009 |
| Auxins | Aquaporin regulation | Paciorek et al. 2005; Péret et al. 2012 |
| Jasmonic acid | Interaction with ABA | Fedoroff, 2002; Fujita et al. 2006; Grant and Jones, 2009; Pieterse et al. 2009; Verma et al. 2016 |
| Ca^2+^ | ABA-mediated activation of guard cell, in concert with other second messengers | Kohler and Blatt, 2002; Verma et al. 2016 |
|  | Second messengers in response to extracellular stimuli | Harper et al. 2004; Ludwig et al. 2004 |
| ROS | Signal to the nucleus | Kirakosyan et al. 2004; Djoukeng et al. 2008; Arbona et al. 2013 |
| Methylglyoxal | Signal factor | Hoque et al. 2012; Schieber and Chandel, 2014; Hasanuzzaman et al. 2017 |
| Proline | Compatible solute, DNA, membrane and protein stabilization. | Arbona et al. 2008 |
|  | ROS scavenger | Arbona et al. 2003; Alet et al. 2012; Sinay and Karuwal, 2014 |
| Polyamines | ROS scavenger | Arbona et al. 2003; Alet et al. 2012; Sinay and Karuwal, 2014 |
|  | Compatible solute; DNA, membrane and protein stabilization. | Arbona et al. 2008 |
| Soluble sugars and polyols | ROS scavenger | Ende and Peshev, 2013; Keunen et al. 2013 |
| Glycine betaine | Osmoregulator, enzymes and protein sterilizer, maintenance of the membrane integrity | Sakamoto and Murata, 2002; Quan et al. 2004 |
